# The role of individual caspases in cell death induction by taxanes in breast cancer cells

**DOI:** 10.1186/s12935-015-0155-7

**Published:** 2015-02-04

**Authors:** Michael Jelínek, Kamila Balušíková, Martina Schmiedlová, Vlasta Němcová-Fürstová, Jan Šrámek, Jitka Stančíková, Ilaria Zanardi, Iwao Ojima, Jan Kovář

**Affiliations:** Department of Cell and Molecular Biology, Third Faculty of Medicine, Charles University, Prague, Czech Republic; Institute of Chemical Biology and Drug Discovery, State University of New York at Stony Brook, Stony Brook, NY USA

**Keywords:** Taxanes, Breast cancer, Caspases, Cell death

## Abstract

**Background:**

In previous study we showed that caspase-2 plays the role of an apical caspase in cell death induction by taxanes in breast cancer cells. This study deals with the role of other caspases. We tested breast cancer cell lines SK-BR-3 (functional caspase-3) and MCF-7 (nonfunctional caspase-3).

**Methods and results:**

Using western blot analysis we demonstrated the activation of initiator caspase-8 and -9 as well as executioner caspase-6 and -7 in both tested cell lines after application of taxanes (paclitaxel, SB-T-1216) at death-inducing concentrations. Caspase-3 activation was also found in SK-BR-3 cells. Employing specific siRNAs after taxane application, suppression of caspase-3 expression significantly increased the number of surviving SK-BR-3 cells. Inhibition of caspase-7 expression also increased the number of surviving SK-BR-3 and MCF-7 cells. On the other hand, suppression of caspase-8 and caspase-9 expression had no significant effect on cell survival. However, caspase-9 seemed to be involved in the activation of caspase-3 and caspase-7. Caspase-3 and caspase-7 appeared to activate mutually. Furthermore, we observed a significant decrease in mitochondrial membrane potential (flow cytometric analysis) and cytochrome c release (confocal microscopy, western blot after cell fractionation) from mitochondria in SK-BR-3 cells. No such changes were observed in MCF-7 cells after taxane treatment.

**Conclusion:**

We conclude that the activation of apical caspase-2 results in the activation of caspase-3 and -7 without the involvement of mitochondria. Caspase-9 can be activated directly via caspase-2 or alternatively after cytochrome c release from mitochondria. Subsequently, caspase-9 activation can also lead to caspase-3 and -7 activations. Caspase-3 and caspase-7 activate mutually. It seems that there is also a parallel pathway involving mitochondria that can cooperate in taxane-induced cell death in breast cancer cells.

## Background

Taxanes are known mitotic poisons. There are two taxanes currently used in cancer therapy, paclitaxel (Taxol®) of natural origin and semi synthetic docetaxel (Taxotere®). They are routinely used in chemotherapy of solid tumors, e.g. breast cancer, ovary cancer, lung cancer and prostate cancer [1]. Unfortunately, resistance of cancer cells to clinically used taxanes (classical taxanes) became a problem. Novel taxanes have been developed in order to overcome resistance of cancer cells [2-4]. Some of these novel taxanes are significantly more effective in resistant cancer cells [5,6].

Taxanes bind to the β subunit of the tubulin heterodimer and prevent depolymerization of microtubules. The stabilization of microtubules blocks progression through the M phase of the cell cycle [7,8]. This state of mitotic arrest normally results in cell death and it is supposedly associated with mitotic catastrophe, which has been observed by many authors in taxanes-treated cells [9-12]. Although there are numerous studies concerning taxane-induced cell death in cancer cells, the molecular mechanism remains elusive [12-14].

It is well known, that functional caspases are required for completing apoptosis after various stimuli. Initiator caspase-9, -8, -10, -2 are involved in apoptosis induction and executioner caspase-3, -6 and -7 are involved in apoptosis execution. The activation of various caspases has been observed after taxane application in many types of cancer cells. The activation of initiator caspase-8, often associated with the death receptor signaling pathway, has been found in cells treated with taxanes [15,16]. In contrast, the role of caspase-8, apart from its involvement in certain amplification loops, has been seriously questioned, particularly in regard to melanoma cancer cells [13,15]. The activity of caspase-10, which, together with caspase-8, is involved in the extrinsic apoptotic pathway, has been observed in human leukemia cells after taxane application. However it was not associated with the activation of death receptors [17].

Caspase-2 is a highly conservative protease and it is known to be involved in cell death induction by several different stimuli, e.g. heat shock, growth factors withdrawal or cytoskeleton damage [18]. It is often activated within a cytoplasmic complex, containing in addition PIDD protein and RAIDD protein, referred to as a PIDDosome [19]. Recently, several laboratories, including ours have reported that caspase-2 appears to play a pivotal role in taxane-induced cell death [13,20,21].

Initiator caspase-9 is involved in the mitochondrial pathway of apoptosis induction and its activity has been found in several cancer cell lines [14,16,22] and also in non-cancer cells [23] after taxane application. It indicates that mitochondria can play an important role in the taxane-induced apoptosis at least in certain cancer cell lines [9,24]. Taxanes have also been found to induce the release of cytochrome c from isolated mitochondria [25] as well as from mitochondria in cancer cells [26,27] or embryonic cells [21]. The release of cytochrome c is a hallmark of apoptosis induction via the intrinsic apoptotic pathway. Another significant feature is decreasing mitochondrial membrane potential (Δψ_m_). Some studies in melanoma and prostate cancer cells have observed decreasing Δψ_m_ after taxane treatment [13,28].

The activation of the key executive caspase-3 and/or cleavage of its substrate PARP have been observed in many cancer cell types after taxane application [12,14,16]. On the other hand, the role of caspase-6 and -7 in cell death induction remains somewhat unclear [17,29]. However, activation of caspase-7 has been detected in breast cancer cells after taxane exposure [30] as well as after combination treatments [31].

In our previous study, we described the activation of caspase-8, -9 and -3 in sensitive and resistant breast cancer cells after apoptosis induction by paclitaxel, the novel taxane SB-T-1216 as well as certain novel fluorinated taxanes. We observed that cytochrome c was released from the mitochondria in one of the tested cancer cell lines. We also showed that caspase-2 was significantly involved in taxane-induced cell death in breast cancer cells [5,12]. Caspase-2 affected the activation of caspase-3, -7, -8 and -9. Thus caspase-2 seemed to play the role of an apical caspase [20].

In the present study we tested the role of individual caspases in taxane-induced cell death in breast cancer cells. We tested two breast cancer cell lines, SK-BR-3 and MCF-7. It is known that functional p53 is absent in SK-BR-3 cells but present in MCF-7 cells. On the other hand, SK-BR-3 cells are known to have functional caspase-3 while MCF-7 cells are deficient in functional caspase-3 [32,33]. Such differences in proteins involved in apoptosis facilitated elucidation of the role of individual caspases in taxane-induced cell death. We found that the activation of apical caspase-2 led to the activation of executioner caspase-3 and −7 without the mitochondria involvement. Although a parallel pathway involving cytochrome c release and caspase-9 activation may also be involved.

## Results

### Effect of taxanes on caspase-3 activation

In order to assess caspase-3 activation in SK-BR-3 cells after taxane application (MCF-7 cells have no functional caspase-3), we tested the time course of procaspase-3 cleavage using western blot analysis.

**Figure 1:**
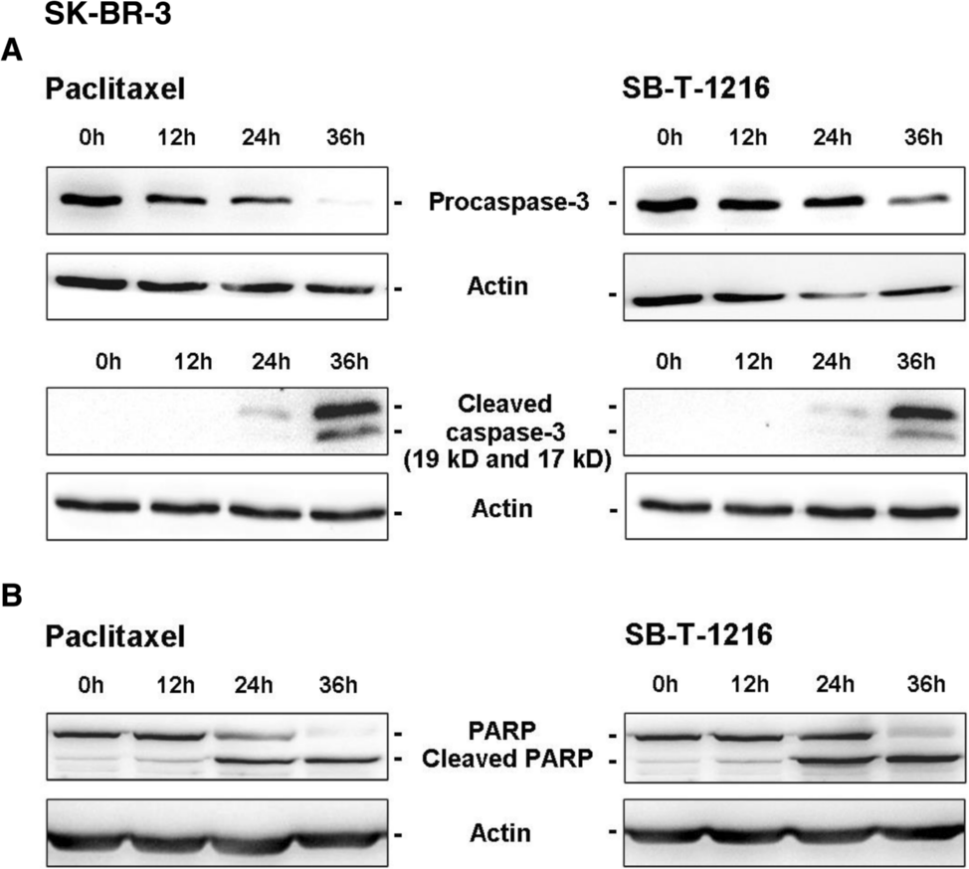
**Effect of paclitaxel and SB** **-T-** **1216 on the activation and activity of caspase-** **3 in SK** **-BR-** **3 cells.** After 0, 12, 24 and 36 h of incubation with paclitaxel or SB-T-1216 (100 nM), levels of **(A)** procaspase-3 and cleaved caspase-3 as well as **(B)** levels of PARP were determined using western blot analysis and relevant antibodies (see “Materials an methods”). Actin levels were used to confirm equal protein loading. The data shown were obtained in one representative experiment of two independent experiments.

Procaspase-3 levels decreased significantly 36 h after application of both taxanes at death-inducing concentration (100 nM in SK-BR-3 cells, see “Materials and methods”). After 48 h, the level of procaspase-3 in SK-BR-3 cells was extremely low (data not shown). The decrease of procaspase-3 levels correlated with dramatically increased levels of the cleaved form of caspase-3 which occurred 36 h after taxane application. However, low levels of cleaved caspase-3 were detectable 24 h after taxanes application (Figure 1A). The time course of caspase-3 activation is in agreement with our finding that most of SK-BR-3 cells are dead before 48 h of taxane treatment (our unpublished data).

Using western blot analysis, the time course of the levels of caspase-3 substrate PARP was also assessed. A significant decrease in PARP levels after 36 h as well as significant increase in cleaved PARP levels after 24 h and 36 h corresponded with caspase-3 activation after taxane application (Figure 1B). In MCF-7 cells, the cleavage of PARP was also detected 36 h after taxane application (data not shown).

### Effect of taxanes on caspase-6 and -7 activations

In order to assess the activation of other executioner caspases (caspase-6 and -7) in both studied cell lines after taxane application, we tested the time course of procaspase-6 and -7 cleavage using western blot analysis.

**Figure 2:**
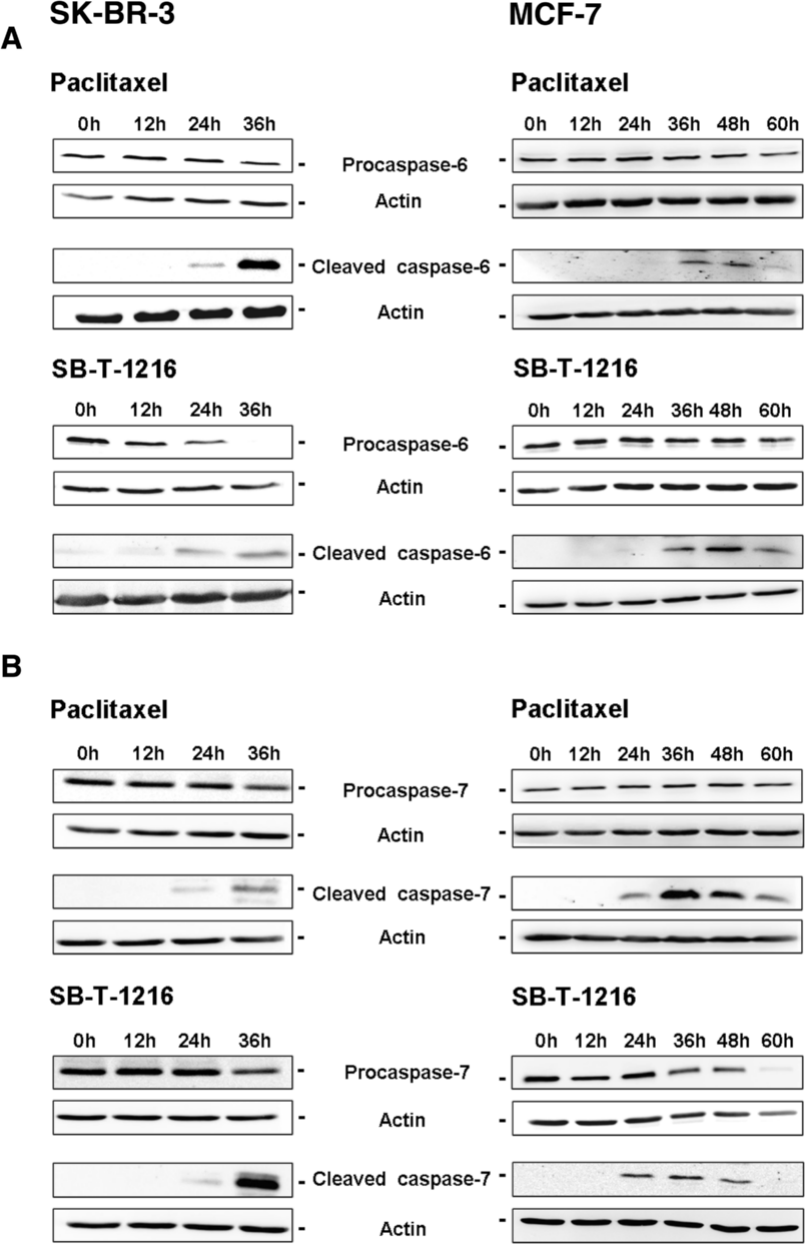
**Effect of paclitaxel and SB** **-T-** **1216 on (A) the activation of caspase**-**6 and (B) the activation of caspase-** **7 in SK**-**BR-** **3 and MCF-** **7 cells.** After 0, 12, 24, 36, 48 and 60 h of incubation with paclitaxel or SB-T-1216 (100 nM for SK-BR-3 cells and 300 nM for MCF-7 cells), levels of procaspases and cleaved caspases were determined using western blot analysis and relevant antibodies (see “Materials and methods”). Actin levels were used to confirm equal protein loading. The data shown were obtained in one representative experiment of two independent experiments.

Procaspase-6 levels decreased somewhat 36 h after the application of taxanes at death-inducing concentration (100 nM) in SK-BR-3 cells. Some decrease was also seen 24 h after the application. Concerning MCF-7 cells, similar effect was seen at least 60 h after taxane application at death inducing concentration (300 nM in MCF-7 cells, see “Materials and methods”). The decrease of procaspase-6 levels in SK-BR-3 cells correlated roughly with increased levels of the cleaved form of caspase-6 24 h and 36 h after taxanes application. In MCF-7 cells, cleaved caspase-6 was seen from 36 h to 60 h after application (Figure 2A).

Procaspase-7 levels decreased slightly 36 h after taxane application in SK-BR-3 cells. In MCF-7 cells, there was also certain decrease in procaspase-7 level 60 h after taxane application. Increased levels of the cleaved form of caspase-7 were detected 24 h and particularly 36 h after taxane application in SK-BR-3 cells and from 24 h to 48 h or even 60 h in MCF-7 cells (Figure 2B). The time course of caspase-6 and -7 activations is also in agreement with our finding that most of cells are dead before 48 h of taxane treatment (our unpublished data).

### Effect of taxanes on caspase-8 and -9 activations

Using western blot analysis, we assessed the activation of initiator caspase-8 and -9 after taxane application by testing the time course of procaspase-8 and -9 cleavages.

**Figure 3:**
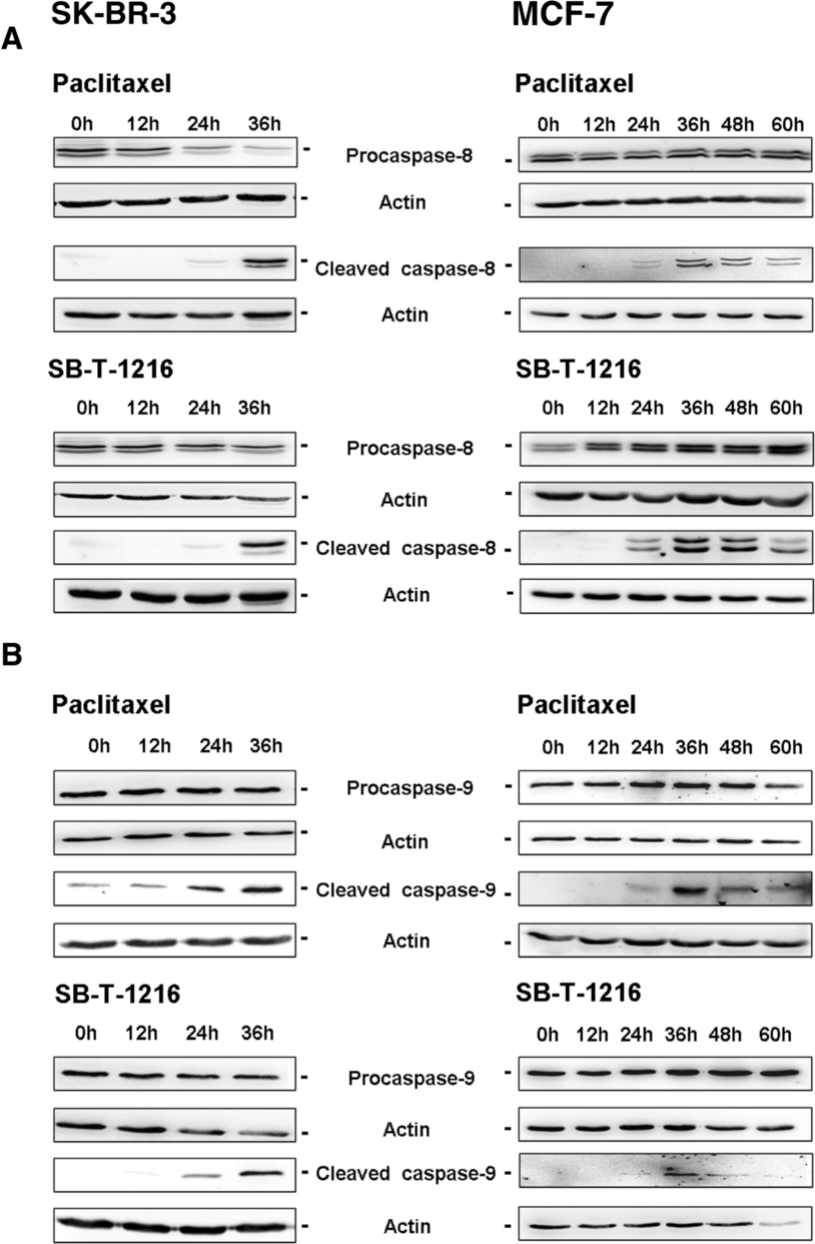
**Effect of paclitaxel and SB** **-T-** **1216 on (A) the activation of caspase** **-8 and (B) the activation of caspase**-**9 in SK** **-BR-** **3 and MCF-** **7 cells.** After 0, 12, 24, 36, 48 and 60 h of incubation with paclitaxel or SB-T-1216 (100 nM for SK-BR-3 cells and 300 nM for MCF-7 cells), levels of procaspases and cleaved caspases were determined using western blot analysis and relevant antibodies (see “Materials and methods”). Actin levels were used to confirm equal protein loading. The data shown were obtained in one representative experiment of two independent experiments.

Procaspase-8 levels decreased 36 h after the application of both taxanes at death-inducing concentration (100 nM) in SK-BR-3 cells. Concerning MCF-7 cells, a decrease in procaspase-8 levels after the application of taxanes at death-inducing concentration (300 nM) was not detected. The decrease of procaspase-8 levels in SK-BR-3 cells correlated with increased levels of the cleaved form of caspase-8 36 h after taxanes application. Cleaved caspase-8 was seen in MCF-7 cells from 24 h to 60 h after taxane application, in spite of the fact that the cleavage of procaspase-8 was not detected here (Figure 3A).

Regarding caspase-9, the decrease of procaspase-9 levels after taxane application was not clearly detected in SK-BR-3 or MCF-7 cells. Increased levels of the cleaved form of caspase-9 appeared 24 h after taxanes application in SK-BR-3 cells. The increase was more pronounced 36 h after the application. Cleaved caspase-9 levels were also seen from 24 h to 60 h after taxane application in MCF-7 cells (Figure 3B). Similarly like in the case of caspase-3, -6 and -7, the time course of caspase-8 and -9 activations is in agreement with our finding that cells are principally dead before 48 h of taxane treatment (our unpublished data).

### Effect of the inhibition of caspase-3, -7, -8 and -9 expression on taxane induced cell death

Employing RNA interference, we assessed the effect of specific inhibition of caspase-3, -7, -8 and -9 expressions on cell death induction by taxane application.

**Figure 4:**
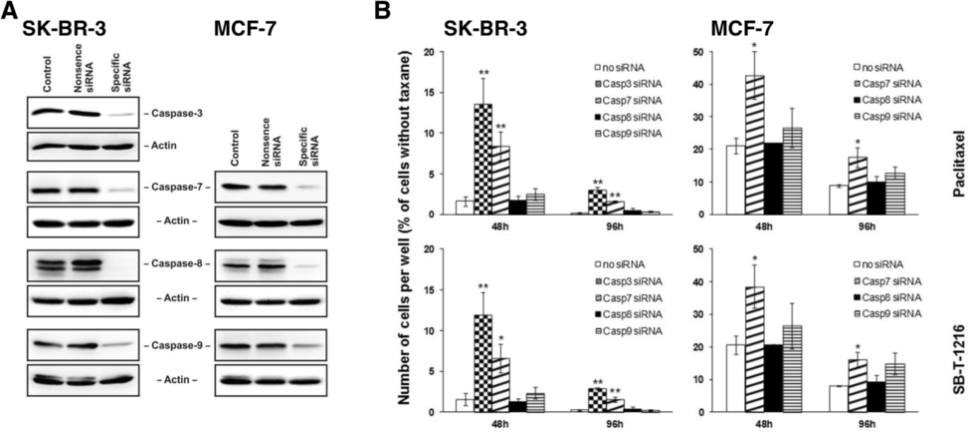
**Effect of inhibition of caspase** **-3,** **-7,** **-8 and -9 expression on cell growth and survival of SK** **-BR-** **3 and MCF-** **7 cells after paclitaxel and SB** **-T-** **1216 application. (A)** Efficiency of caspase suppression based on specific siRNAs in SK-BR-3 and MCF-7 cells is shown. Levels of caspases were determined using western blot analysis and relevant antibodies (see “Materials and methods”). Actin levels were used to confirm equal protein loading. **(B)** The effect of specific caspase siRNAs on the growth and survival of SK-BR-3 and MCF-7 cells after taxane treatment (100 nM for SK-BR-3 cells and 300 nM for MCF-7 cells) is presented. The cells were seeded at 20 × 10^3^ cells/100 μl of medium per well and prepared as described (see “Materials and methods”). After 48 and 96 h of incubation, the number of living cells was determined (see “Materials and methods”). Each column represents the mean of 3 or 4 separate cultures ± SEM. *P < 0.05, **P < 0.01 when comparing the number of living cells in cultures with individual specific siRNAs and culture with no siRNA.

First, the efficiency of the RNA interference was tested. It revealed that inhibition of the expression of individual caspases was efficient enough in both SK-BR-3 and MCF-7 cells (Figure 4A). Furthermore, nonsense siRNA or specific caspase siRNAs did not significantly affect cell growth or survival in either cell line. Cell transfected with siRNAs seemed to grow slightly slower when compared with control cells (data not shown). In spite of this fact, in the case of caspase-3 and -7 we were able to detect significant increase of cell growth and survival after the inhibition of caspase expression by siRNA application when cells were incubated with taxanes (see below).

After 48 h of incubation with taxanes at death-inducing concentrations (100 nM), inhibition of caspase-3 expression (81%) resulted in an approximately 6-fold increase in the number of surviving SK-BR-3 cells. For both taxanes, it represents a statistically significant increase from about 2% to 12-14% of the number of cells cultured without taxane. The inhibition of caspase-7 expression (81%) resulted in an approximately 4-fold increase in the number of surviving SK-BR-3 cells after 48 h of incubation. It again represents a statistically significant increase (from about 2% to 7-8%). After 96 h, the effect of the inhibition of caspase-3 and caspase-7 expression was similar or even more pronounced. On the other hand, we did not detect any significant effect of caspase-8 (94%) or caspase-9 (70%) suppression on cell death induction by taxanes in SK-BR-3 cells (Figure 4B).

Concerning MCF-7 cells, inhibition of caspase-7 expression (81%) increased the number of surviving cells by approximately 2-fold after 48-h incubation with taxanes at death-inducing concentration (300 nM). It was a statistically significant increase from about 20% to about 40% of the number of cells cultured without taxane. After 96 h, the effect of the inhibition of caspase-7 expression was very similar. We did not detect a significant effect of caspase-8 (89%) and caspase-9 (61%) suppression on taxane-induced cell death in MCF-7 cells. Perhaps there was a slight, although insignificant, increase of the number of surviving cells linked to the inhibition of caspase-9 expression (Figure 4B).

### Effect of the inhibition of caspase-8, -9, -3 and -7 expression on taxane induced activation of caspase-8, -9, -3, -7

**Figure 5:**
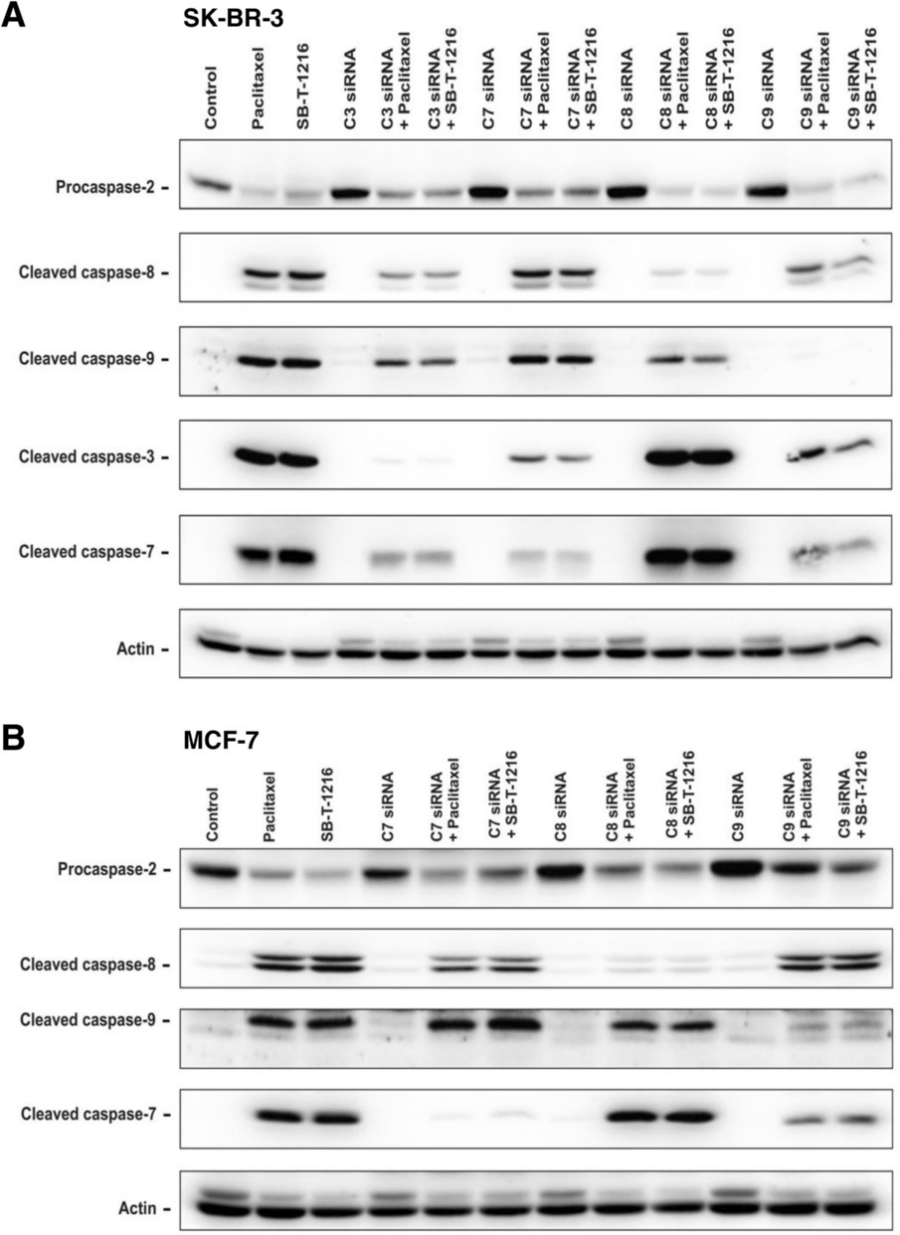
**Effect of the inhibition of caspase-** **3,** **-7,** **-8 and -9 expression on the activation of caspase** **-3,** **-7,** **-8 and** **-9 in (A) SK**-**BR-** **3 and (B) MCF**-**7 cells after paclitaxel and SB** **-T-** **1216 application.** Specific caspase-3 siRNA, caspase-7 siRNA, caspase-8 siRNA and caspase-9 siRNA were used. Control cells were incubated without taxane and siRNA. After 36 h of incubation with the tested taxane (100 nM for SK-BR-3 cells and 300 nM for MCF-7 cells) and relevant specific siRNA, levels of cleaved caspases were determined using western blot analysis and relevant antibodies (see “Materials and methods”). Actin levels were used to confirm equal protein loading. The data shown were obtained in one representative experiment of two independent experiments.

Using the siRNA technique, we assessed the effect of specific inhibition of caspase-3, -7, -8 and -9 expression on the activation of caspase-8, -9, -3, and -7 after taxane application. Inhibition efficiency of individual caspases is mentioned above (or see “Materials and methods”). To confirm caspase-2 role as an apical caspase [20], the effect of specific inhibition of the expression of tested caspases on procaspase-2 cleavage was also assessed after taxane application. No significant effect was found (Figure 5).

After 36-h incubation of SK-BR-3 cells with taxanes at death inducing concentration (100 nM), subsequent western blot analysis showed significantly decreased cleavage of caspase-8, -9 and -7 due to inhibition of caspase-3 expression. Decreased cleavage of caspase-3 was observed in response to inhibition of caspase-7 expression. Decreased cleavage of caspase-9 was seen in response to inhibition of caspase-8 expression and decreased cleavage of caspase-3 and -7 linked to inhibition of caspase-9 expression (Figure 5A).

Concerning MCF-7 cells after 36 h of incubation with taxanes at death-inducing concentration (300 nM), inhibition of caspase-7 expression did not significantly affect the cleavage of caspase-9 but it slightly affected the cleavage of caspase-8. Inhibition of caspase-8 expression did not affect the cleavage of caspase-9 and -7. Inhibition of caspase-9 expression did not affect the cleavage of caspase-8, but it significantly decreased the cleavage of caspase-7 (Figure 5B).

### Effect of taxanes on mitochondrial membrane potential (Δψ_m_)

We employed [DiOC6(3)] staining and subsequent flow cytometry to assess the effect of taxanes on mitochondrial membrane potential (Δψ_m_).

**Figure 6:**
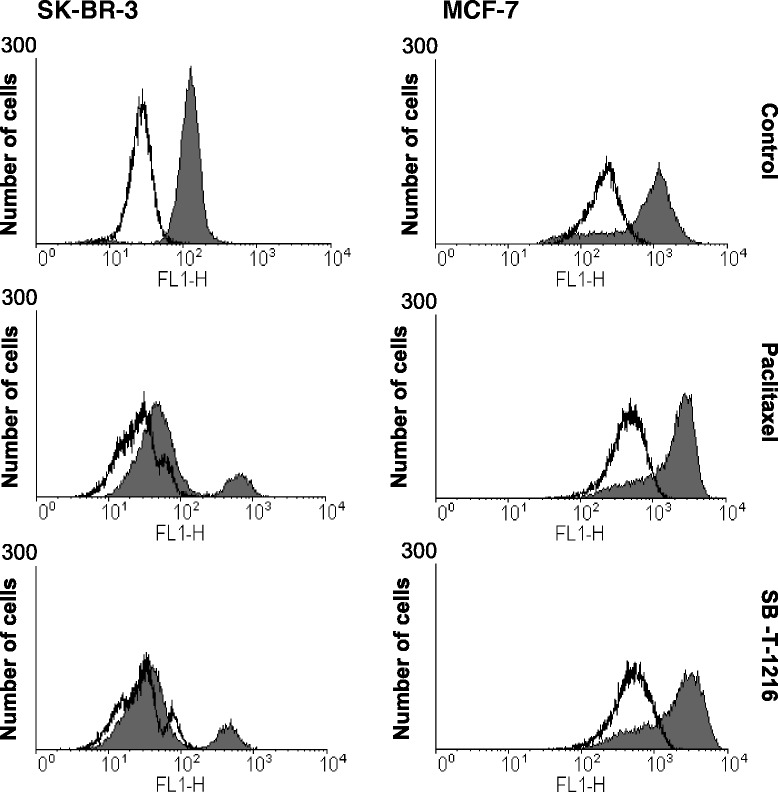
**Effect of paclitaxel and SB** **-T-** **1216 on the mitochondrial membrane potential** **(Δψ**_**m**_ **) in SK** **-BR** **-3 and MCF-** **7 cells.** Control cells were incubated without taxane. After 36 h (SK-BR-3) or 60 h (MCF-7) of incubation with tested taxane (100 nM for SK-BR-3 and 300 nM for MCF-7) the mitochondrial membrane potential was assessed using flow cytometry analysis of cells after staining with [DiOC6(3)] (see “Materials and methods”). The collapse of Δψ_m_ caused by the application of CCCP is shown (blank areas). The data shown were obtained in one representative experiment of three independent experiments.

In SK-BR-3 cells, within 36 h of incubation with taxanes at death-inducing concentration (100 nM), Δψ_m_ decreased significantly close to a total collapse. On the other hand, Δψ_m_ did not show any significant decrease in MCF-7 during 60 h after taxane application. It clearly demonstrated different responses of mitochondrial membrane potential to taxane application in SK-BR-3 and MCF-7 cells (Figure 6).

### Effect of taxanes on cytochrome c release

The effect of tested taxanes on cytochrome c release from mitochondria was assessed using confocal microscopy and cell fractionation followed by western blot analysis.

**Figure 7:**
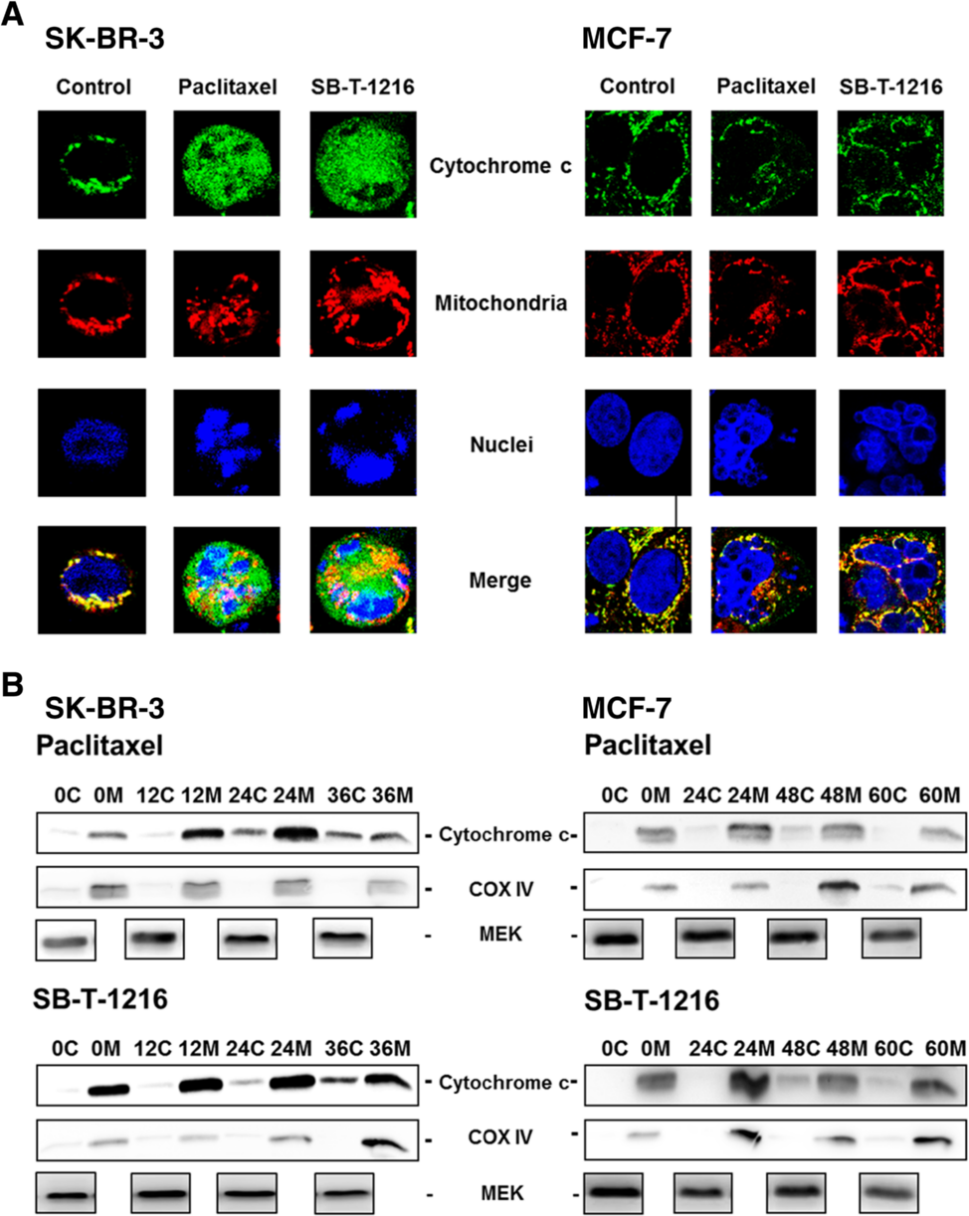
**Effect of paclitaxel and SB-** **T-** **1216 on cytochrome c release from mitochondria in SK** **-BR-** **3 and MCF** **-7 cells. (A)** After 36 h of incubation with tested taxane (100 nM for SK-BR-3 cells and 300 nM for MCF-7 cells), the localization of cytochrome c within the cells was detected using confocal microscopy (see “Materials and methods”). Control cells were incubated without taxane. The localization of cytochrome c (green), mitochondria (red), nuclei (blue) and the merge of cytochrome c, mitochondria and nuclei are shown. The data shown were obtained in one representative experiment of three independent experiments. **(B)** After 0, 12, 24, 36, 48 and 60 h of incubation with tested taxane (100 nM for SK-BR-3 cells and 300 nM for MCF-7 cells), levels of cytochrome c in mitochondrial (M) and cytosolic (C) fractions were determined using western blot analysis and relevant antibodies (see “Materials and methods”). COX IV (integral mitochondrial protein) level was used to confirm proper fractionation. MEK levels were used to confirm equal protein loading of cytosolic fraction. The data shown were obtained in one representative experiment of three independent experiments.

Confocal microscopy showed that cytochrome c was mainly localized in the mitochondria of control SK-BR-3 as well as MCF-7 cells. After 36 h of incubation of SK-BR-3 cells with taxanes at death-inducing concentration (100 nM), cytochrome c was released from the mitochondria into the cytosol. However, in MCF-7 cells, cytochrome c was still found within mitochondria 36 h after taxane application (300 nM) (Figure 7A).

These results were confirmed using western blot analysis after cell fractionation. Cytochrome c was shown to be mainly localized in the mitochondrial cell fraction in both SK-BR-3 and MCF-7 cells before taxane application. In SK-BR-3 cells, cytochrome c levels increased rapidly in the cytosolic fraction 24 h and particularly 36 h after taxane application. In MCF-7 cells, cytochrome c stayed mainly in the mitochondrial fraction after taxane application. Thus, after taxane application, cytochrome c is released from mitochondria only in SK-BR-3 cells, but not in MCF-7 cells (Figure 7B).

## Discussion

In our previous paper [20] we studied the role of caspase-2 in taxane-induced cell death in breast cancer cells. It was suggested that caspase-2 plays the role of an apical caspase. This study deals with the role of other caspases, i.e. initiator caspase-8 and -9 as well as executioner caspase-3 and -7 in taxane-induced cell death in breast cancer cells. The relationship between activation of individual caspases was of particular interest. We used two breast cancer cell lines, SK-BR-3 (nonfunctional p53, functional caspase-3) and MCF-7 (functional p53, nonfunctional caspase-3) as an experimental model. We previously reported that p53 is activated in MCF-7 cells after taxane application. However, we did not observed p53-induced increased expression of PIDD protein or any other function of p53 in activation of caspase-2 that plays important role in cell death induction [20]. The role of p53 in apoptosis induction after taxane application is also questioned because we did not find the activation of mitochondrial death pathway to have a decisive role in cell death induction here. Importantly, apoptosis was induced in both tested cell lines even SK-BR-3 have no functional p53 [20]. Taken together, we do not suppose p53 to play an important role in cell death induction after taxane application in tested breast cancer cells.

We tested two taxanes, classical (clinically used) paclitaxel and novel taxane SB-T-1216. The effect of both taxanes was found to be very similar.

We found that caspase-8 was activated after taxane application at death-inducing concentrations in both tested cell lines (see Figure 3). However, the suppression of caspase-8 expression did not affect cell survival after taxane treatment at all (see Figure 4). Activation of other caspases, especially the executioner caspases, was not significantly affected in cells where caspase-8 expression had been inhibited (see Figure 5). The role of caspase-8 in various types of cancer cells after taxane treatment has been previously discussed in several reports [12,15]. Interestingly, caspase-8 activation was usually observed only with the simultaneous activation of other initiator caspases (caspase-2 and caspase-9) after taxane application [12,16,22]. The role of caspase-8 in taxane-induced cell death was questioned by Park et al. [17]. They did not find any significant role of caspase-8 in taxane-induced cell death in lymphoid cells. Similarly, we suggest that caspase-8 does not play any significant role in cell death induction by taxanes in breast cancer cells.

**Figure 8:**
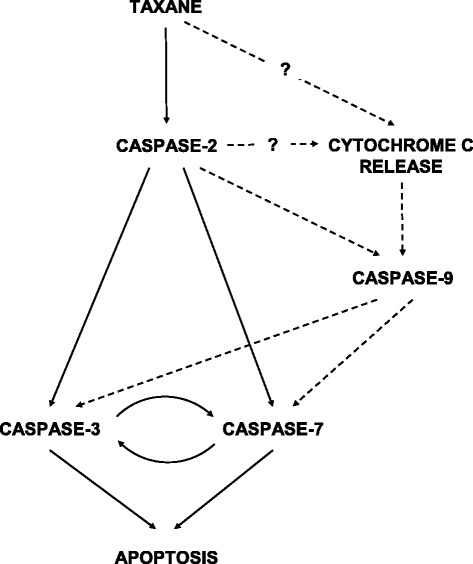
**Scheme showing suggested pathways leading to the activation of individual caspases during apoptosis induction by taxanes in breast cancer cells.** Solid lines represent the main pathway of apoptosis induction and dashed lines represent parallel pathways.

On the contrary, caspase-2 seems to play a key role in cell death induction by taxanes [20]. We and others have shown that cancer as well as non-cancer cells without functional caspase-2 were more resistant to taxanes [13,20,21]. We showed that the inhibition of caspase-2 expression resulted in decreased activation of caspase-9, -3 and -7 after taxane application in breast cancer cells which implies that caspase-2 plays the role of an apical caspase in these cells [20] (see Figure 8).

Taxanes have been shown to induce the mitochondrial pathway of apoptosis induction by several authors [5,9,23]. Also in this study, we observed significant decreases of Δψ_m_ and cytochrome c release from mitochondria in SK-BR-3 cells after taxane application. However, we did not detect any change in Δψ_m_ or cytochrome c release in taxane-treated MCF-7 cells (see Figures 6 and 7). Previous reports have shown that cytochrome c was released from mitochondria and Δψ_m_ decreased in certain types of cancer cells treated with taxane [5,27]. On the other hand, other types of cancer cells underwent cell death without cytochrome c release from mitochondria [5]. Recently, the role of cytochrome c in taxane-induced cell death was also assessed by Li et al. [34]. They observed cytochrome c release from mitochondria but cell death was induced without cytochrome c involvement. Thus, it seems that the mitochondrial pathway does not play an essential role in apoptosis induction by taxanes, at least in certain types of cancer cells (see Figure 8).

Caspase-9 was activated in SK-BR-3 cells and a relatively low degree of activation was also found in MCF-7 cells (see Figure 3). Activation of caspase-9 has been observed by many authors in various types of cancer cells after taxane treatment and it usually appeared together with caspase-3 activation [12,16,35]. Concerning the mechanism of caspase-9 activation following taxane treatment, we suggest that there are at least two pathways involved. Caspase-9 can be activated in the cytosol via the classical pathway after cytochrome c release from mitochondria or it can be activated via caspase-2 activation without the involvement of cytochrome c (see Figure 8). Although cells were slightly protected against cell death induction by the inhibition of caspase-9 expression, the effect on cell survival after taxane application was not significant in both tested cell lines (see Figure 4). Independence of cell death induction by taxanes in MCF-7 cells on caspase-9 activity was described previously [36]. Using caspase-9 siRNA, we also found some decreased activation of executioner caspase-3 and -7 after taxane application (see Figure 5). Finally, we suggest that caspase-9 could play a role in the parallel pathway of executioner caspase-3 and -7 activations after taxane application (see Figure 8).

Caspase-3 was activated and its substrate PARP was cleaved in SK-BR-3 cells after taxane application (see Figure 1). Caspase-3 was also found to be activated in other cancer cell types after exposure to paclitaxel [16,24,37] or docetaxel [38]. Inhibition of caspase-3 expression increased the number of surviving SK-BR-3 cells significantly (see Figure 4). Concerning MCF-7 cells, there are previous reports demonstrating that overexpression of caspase-3, after transfection of the caspase-3 gene, increased the sensitivity of cells to paclitaxel [32]. On the other hand, caspase-3-independent cell death has been also reported to occur in MCF-7 cells transfected by the caspase-3 gene after paclitaxel application [36]. We also demonstrated that the suppression of caspase-3 expression decreased the activation of caspase-7 (see Figure 5). It confirms that caspase-7 is a caspase-3-activated caspase. Thus, caspase-3 seems to be a universally important executioner caspase for taxane-induced cell death in a variety of cancer cell types.

Executioner caspase-7 and caspase-6 were activated in tested cell lines after taxane application (see Figure 2). Caspase-7 activation was also observed during cell death induction by zoledronic acid in breast cancer cells [39] or in other types of cancer cells after paclitaxel application [24,30]. We showed that the suppression of caspase-7 expression significantly increased the number of surviving SK-BR-3 as well as MCF-7 cells (see Figure 4). MCF-7 cells without functional caspase-7 have been recently described to be more resistant to paclitaxel [40]. We also demonstrated that the inhibition of caspase-7 expression led to a decrease of caspase-3 activation in SK-BR-3 cells (see Figure 5). Thus it seems that caspase-3 and -7 cooperate in the later phases of apoptosis induction by taxanes via their mutual activation (see Figure 8). Cooperation of caspase-3 and caspase-7 during cell death induction was recently described by Brentnall et al. [41].

## Conclusion

We can summarize that caspase-2 plays the key role of an apical caspase in main death-inducing pathway after taxane application in tested breast cancer cells. This pathway leads from activated caspase-2 to the activation of executioner caspase-3 and -7 without the involvement of mitochondria. There is mutual activation of caspase-3 and -7. Furthermore, taxanes appear to be able to induce cytochrome c release from mitochondria in some breast cancer cells. In these cases caspase-9 is activated. However, caspase-9 can also be activated here without the mitochondrial involvement, probably via caspase-2 activation. This parallel pathway represents another possibility for caspase-3 and caspase-7 activation. It suggests that both pathways can cooperate in cell death induction by taxanes in at least some types of breast cancer cells.

## Materials and methods

### Materials

Paclitaxel was obtained from Sigma-Aldrich (St. Louis, MO, USA). SB-T-1216 was synthesized at the Institute of Chemical Biology and Drug Discovery (Stony Brook, NY, USA). Taxanes were dissolved in DMSO (tissue culture quality) to obtain a 1 mM stock solution.

For western blot analysis, the following primary antibodies were used: mouse monoclonal antibody against caspase-3 (#9668), rabbit polyclonal antibody against cleaved caspase-3 (#9661), rabbit polyclonal antibody against caspase-6 (#9762), rabbit polyclonal antibody against cleaved caspase-6 (#9761), rabbit polyclonal antibody against caspase-7 (#9492), rabbit polyclonal antibody against cleaved caspase-7 (#9491), mouse monoclonal antibody against caspase-8 (#9746), rabbit monoclonal antibody against cleaved caspase-8 (#9496), rabbit polyclonal antibody against caspase-9 (#9502), rabbit polyclonal antibody against cleaved caspase-9 (#9505), rabbit polyclonal antibody against COX IV (#4844), rabbit polyclonal antibody against cytochrome c (#4272), rabbit polyclonal antibody against PARP (#9542) from Cell Signaling Technology (Danvers, MA, USA), and mouse monoclonal antibody against actin (AC-40, A3853) from Sigma-Aldrich.

Casp3 Silencer® Select Validated siRNA 4427038, Casp7 Silencer® Select Validated siRNA 4427038, Casp8 Silencer® Select Validated siRNA 4427038, and Casp9 Silencer® Select Validated siRNA 4427037 were from Life technologies (Carlsbad, CA, USA).

### Cells and culture conditions

Human breast carcinoma cell lines SK-BR-3 and MCF-7 were obtained from American Type Culture Collection (ATCC, Rockville, MD, USA) and the National Cancer Institute (Frederick, MD, USA), respectively. The cells were maintained in a culture medium at 37°C in a humidified atmosphere of 5% CO_2_ in air. The culture medium consisted of basic medium supplemented with 10% heat-inactivated fetal bovine serum (Biochrom AG, Berlin, Germany). The basic medium was RPMI 1640 medium (Sigma-Aldrich, St. Louis, MO, USA) containing extra L-glutamine (300 μg/ml), sodium pyruvate (110 μg/ml), HEPES (15 mM), penicillin (100 U/ml) and streptomycin (100 μg/ml) [42]. For experiments, paclitaxel and SB-T-1216 were diluted in culture medium to produce a final concentration of 100 nM (SK-BR-3) and 300 nM (MCF-7). These cell death inducing concentrations are the lowest taxane concentrations with nearly maximum effect on tested cells. The concentrations were derived from dose response experiments described in detail previously [20]. DMSO was used as a dissolvent agent for taxanes. DMSO itself at used concentrations was found without any effect on tested cells.

### Assessment of cell growth and survival

Cells were harvested and seeded at 20 × 10^3^ cells/100 μl of culture medium into the wells of a 96-well plastic plate. After a 24-h preincubation period allowing cells to attach, the culture medium was replaced by either culture medium without taxane (control) or medium with one of tested taxanes (paclitaxel or SB-T-1216) at desired concentrations. Cell growth and survival were evaluated after 48 h and 96 h of incubation. The number of living cells was determined using a hemocytometer after staining with trypan blue.

### Preparation of cell lysates

Cells at desired concentrations were seeded into wells of a plastic plate, Petri dishes or culture flasks and taxanes were applied after a 24-h preincubation. After the incubation period, cells were harvested by low-speed centrifugation (2000 rpm, 9 min, 4°C), washed in PBS and centrifuged. Cell pellets were stored at -80°C. Frozen pellets were resuspended in RIPA buffer (Sigma Aldrich) containing a 1% mixture of protease inhibitors P8340 (AEBSF 104 mM, Aprotinin 80 μM, Bestatin 4 mM, E-64 1.4 mM, Leupeptin 2 mM, Pepstain A 1.5 mM, Sigma Aldrich). Protein lysates were centrifuged (14,000 rpm, 20 min, 4°C) and the supernatants containing proteins were stored at -80°C. Protein lysates were than analyzed using western blot.

### Cell fractionation

Cells (approximately 3.6 × 10^6^ cells per sample) were seeded into Petri dishes or culture flasks and taxanes were applied after a 24-h preincubation. After the incubation period, cells were harvested by low-speed centrifugation (2000 rpm, 9 min, 4°C), washed in PBS and centrifuged again. Cell pellets were resuspended in a specific lysis buffer (75 mM NaCl, 1 mM NaH_2_PO_4_, 8 mM Na_2_HPO_4_, 250 mM sucrose and 1% mixture of protease inhibitors P8340 from Sigma Aldrich) containing 0.635 mM digitonin D141 (Sigma-Aldrich) and vortexed for 30 s. Lysates were than centrifuged (14,000 rpm, 1 min, 4°C) and supernatants (cytosolic fractions) were removed and stored at -80°C. The specific lysis buffer described above containing 6.35 mM digitonin D141 (Sigma-Aldrich) was added to the pellets and suspensions were vortexed for 30 s and centrifuged (14,000 rpm, 1 min, 4°C). After centrifugation, supernatants (mitochondrial fractions) were removed and stored at −80°C. Cell fractions were analyzed using western blot.

### Western blot analysis

First, the concentration of proteins in cell lysates was assessed using BCA Protein Assay Reagent from Pierce (Thermo Fisher Scientific, Rockford, IL, USA).

Depending on protein concentration, cell lysates were diluted in RIPA buffer to the gel-loading concentration of proteins (2.5 μg/μl), mixed with equal volumes of sample buffer (0.125 M Tris/HCl pH 6.8, 10% glycerol, 4% SDS, 0.25 M DTT) and heated for 5–7 min at 110°C. Protein samples were separated using a protein electrophoresis (Bio-Rad, Hercules, CA). Proteins separated by SDS-PAGE were blotted onto 0.2 μm nitrocellulose membrane PROTRAN BA 83 (Whatman-Schleicher and Schuell, Maidstone, UK) for 3 h at 0.25 A, using a MiniProtean II blotting apparatus (Bio-Rad). The membrane was blocked with 5% non-fat dry milk or 5% BSA in TBS for 15–20 min and incubated with the primary antibody at 4°C overnight. After the incubation, the membrane was washed three times (5–10 min) with TBS containing 0.1% Tween-20. Then it was incubated for 1–2 h with the corresponding horseradish peroxidase-conjugated secondary antibody (Santa Cruz Biotechnology, Santa Cruz, CA, USA). Afterward, the membrane was washed (as described above) and the chemiluminescence signal was detected using the Supersignal reagents from Pierce (Thermo Fisher Scientific) and a CCD device (Kodak).

### RNA interference

Based on the manufacturer’s instructions (INTERFERin™ in vitro siRNA Transfection Protocol, Polyplus transfection™), RNA interference in SK-BR-3 and MCF-7 cells was completed. The cells were seeded at 2.1 × 10^5^ cells/6 ml of culture medium into a Petri dish for a 24-h preincubation. The siRNAs (see “Materials”) were diluted in 400 μl OPTI-MEM® I Reduced Serum Medium (Gibco, Invitrogen™ Life Technologies, Carlsbad, CA, USA) to a final concentration of 5 nM and the INTERFERin™ transfection agent (18 μl per reaction mixture) was added. The mixture was intensively vortexed and incubated for 10 min at room temperature to form a transfection complex. The preincubation medium in Petri dish was replaced by 4 ml of fresh culture medium. Transfection mixture was added and gently mixed. Cells were incubated in the presence of transfection complexes for 72 h. After incubation, cells were harvested into fresh culture medium and seeded at 2 × 10^5^ cells/ ml for further analysis. The efficiency of gene silencing using RNA interference was confirmed using western blot analysis followed by densitometry: in the case of SK-BR-3 cells 81% for caspase-3, 81% for caspase-7, 94% for caspase-8 and 70% for caspase-9, and in the case of MCF-7 cells 81% for caspase-7, 89% for caspase-8 and 61% for caspase-9.

### Flow cytometric analysis of the mitochondrial membrane potential (Δψ_m_)

Cells (approximately 5 × 10^5^ cells per sample) were seeded into Petri dishes and taxanes were applied after a 24-h preincubation. After the incubation period, cells were harvested by low-speed centrifugation (2000 rpm, 9 min, 4°C) and resuspended in PBS. To assess Δψ_m_, cells were incubated with 20 nM 3, 3′-dihexyloxacarbocyanine iodide [DiOC6(3)] from Invitrogen (Grand Island, USA) at 37°C for 20 min. As a negative control, the cells were pre-incubated with 100 μM carbonyl cyanide m-chlorophenylhydrazone (CCCP) (Sigma-Aldrich, St. Louis, USA), a protonophore causing a complete disruption of the Δψ_m_, at 37°C for 20 min. After incubation, samples were kept on ice. The fluorescence of cells was measured using a FACS Calibur cytometer (Becton Dickinson, San Jose, CA, USA).

### Confocal microscopy

Confocal microscopy was described previously [43]. Briefly, cells were seeded onto coverslips (approximately 2 × 10^5^ cells per coverslip) and taxanes were applied after 24 h of preincubation. After 36 h of incubation, cells were stained with Mitotracker Red 480 (Molecular Probes, Grand Island, USA), fixed with 4% paraformaldehyde at 37°C for 15 min and permeabilized with 0.1% Triton X-100 in 4% paraformaldehyde for the next 15 min. After washing with PBS, cells were blocked with Image-iT™ FX signal enhancer (Molecular Probes, Invitrogen, Eugene, OR, USA) for 30 min. Next, cells were stained with the corresponding primary antibody at 4°C overnight. Cells were than washed with PBS and incubated with the corresponding secondary antibody for 1 hour in the dark at room temperature. Finally, cells were washed again with PBS. Stained cells on coverslips were transferred onto a droplet of Vectashield® Mounting Medium with DAPI (Vector Laboratories, Burlingame, CA, USA) and sealed. Samples were analyzed using a Leica TCS SP5 confocal microscope (Bannockburn, IL, USA) with relevant excitation and emission wavelengths.

### Statistical analysis

Statistical significance of differences was determined using the Student’s t-test. P < 0.05 and P < 0.01 were considered statistically significant at the 5% and 1% levels, respectively.
